# Brain damage resembling acute necrotizing encephalopathy as a specific manifestation of haemophagocytic lymphohistiocytosis - induced by hypersensitivity

**DOI:** 10.1186/s13052-016-0286-z

**Published:** 2016-08-31

**Authors:** Dongling Dai, Feiqiu Wen, Sixi Liu, Shaoming Zhou

**Affiliations:** 1 First Affiliated Hospital of Jinan University, No.601 Huangpu Avenue West, Guangzhou, 510630 China; 2 Shenzhen Children’s Hospital, 7019, Yitian Road, Shenzhen, 518036 Futian District China

**Keywords:** Acute necrotizing encephalopathy of childhood, Brain damage, Haemophagocytic lymphohistiocytosis, Hypersensitivity

## Abstract

**Background:**

Both haemophagocytic lymphohistiocytosis and acute necrotizing encephalopathy are life-threatening condition. It presents major diagnostic difficulties, since it may have a diversity in clinical picture and with many conditions leading to the same clinical presentation. So it is key important to understand the disorders.

**Case presentation:**

We report a pediatric case of haemophagocytic lymphohistiocytosis with specific presentation which predominantly featured as acute necrotizing encephalopathy of childhood. We discuss the diagnosis and differential diagnosis, and speculate the etiology of haemophagocytic lymphohistiocytosis is due to hypersensitivity.

**Conclusion:**

Haemophagocytic lymphohistiocytosis and brain damage in this case may be induced by hypersensitivity, which have good clinical outcome if diagnosed and treated early.

## Background

Hemophagocytic lymphohistiocytosis (HLH) was first reported by Risdall et al. [1]. It is a life-threatening condition that can rapidly deteriorate and lead to multiple organ failure and death [2]. Cardinal symptoms of HLH are prolonged fever, hepatosplenomegaly and cytopenias, and characteristic laboratory findings in HLH include hyperferritinemia, hypertriglyceridemia, cytopenia, hypoalbuminemia, decreased fibrinogen, hypercholesterolemia, and abnormal liver function [3].

Acute necrotizing encephalopathy of childhood (ANEC) represents an entity of acute encephalopathy which manifests with symptoms of coma, convulsions, and hyperpyrexia [4]. This disease is associated with severe neurological sequelae and profoundly high mortality [5]. ANEC is characterized by multiple and symmetrical lesions with edema and necrosis in the thalamus, the cerebral and cerebellar medulla, and the brainstem tegmentum [4].

Haemophagocytic lymphohistiocytosis and acute necrotizing encephalopathy of childhood are life-threatening conditions with severe consequences and high mortality. It is key important to understand the disorders. The present paper describes the case of a 3-month-old boy presenting with characteristic manifestation resembling acute necrotizing encephalopathy of childhood associated with hemophagocytic lymphohistiocytosis, which was thought due to allergic reaction.

## Case presentation

The 3-month-old boy, the only child of non-consanguineous parents, was hospitalized in Shenzhen Children's Hospital because of persistent fever and skin rash for more than two weeks, resistant to conventional therapy. He had no cough, vomiting, lethargy or irritability. His past history included allergic rashes, and his mother suffered allergic reaction. There was no HLH patient in his family.

**Fig. 1:**
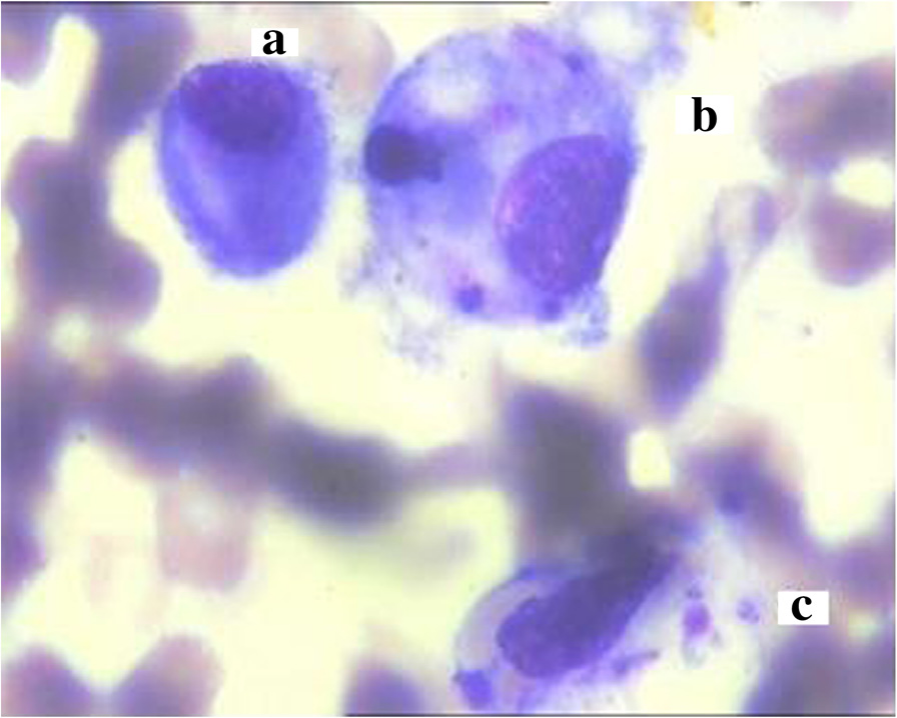
Hemophagocytic histiocytes in bone marrow. Bone marrow aspiration showing a normal histiocyte (**a**), a hemophagocytic histiocyte containing aphagocytosed neutrophil and platelets (**b**), a hemophagocytic histiocyte containing aphagocytosed erythroblast and platelets (**c**). (Wright staining; ×1000)

**Fig. 2:**
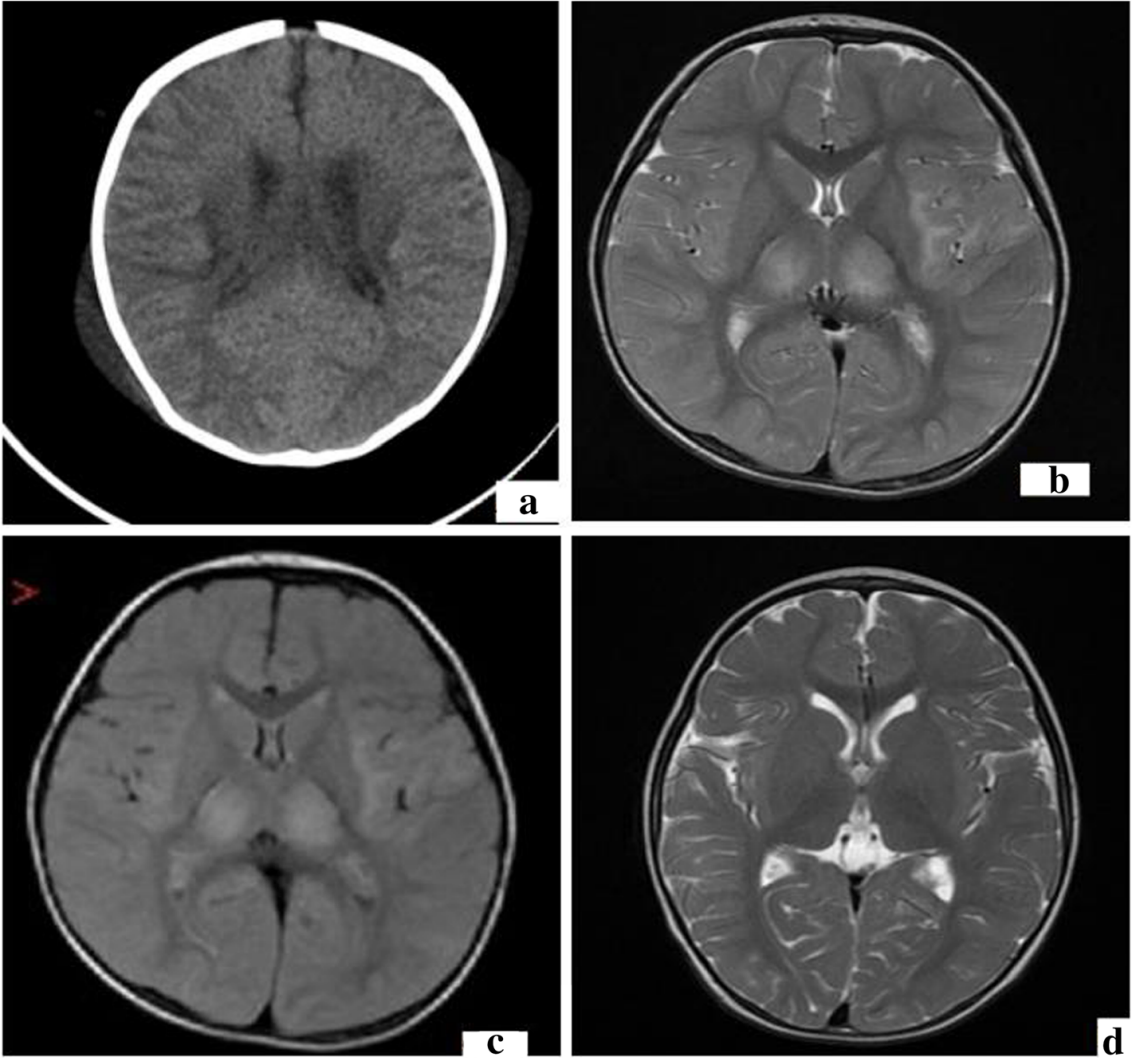
Neurological images. Brain magnetic resonance imaging (MRI) on admission revealed extensive edema (**a**); after 4 weeks, it showed long T2 signal (**b**) and hyperintensity on fluid-attenuated inversion recovery (FLAIR, **c**) mainly in thalamus, basal ganglia, brainstem. 1 year later, the neurological image (MRI) showed slightly abnormal shape of bi-lateral ventricular, not any other obvious abnormal changes noticed (**d**)

On admission, physical examination found rashes mainly in face, trunk and limbs, liver and spleen just palpable, no edema of palms, no periungual or perianal desquamation, no cervical lymphadenopathy, no conjunctival congestion, no strawberry tongue, erythema of the oropharyngeal mucosa or cracking of the lip. No positive neurological sign was found. Laboratory tests showed an increase in leukocytes (20.1 × 10^9^/L) and neutrophils (12.82 × 10^9^/L), erythrocyte sedimentation rate (33 mm/h), positive antinuclear antibody and antineutrophil cytoplasmic antibody, mild anemia (hemoglobin 89 g/L) and slightly elevation of C-reactive protein (19.5 mg/L). Liver function, lymphocyte subtypes, immunoglobulin isotypes, serum ferritin, coagulation function test and blood fat level were normal; Serology for human immunodeficiency virus; widal test; bacterial culture of peripheral blood, bone marrow and urine; hepatitis A, B, and C; mycoplasma pneumoniae (IgM and DNA); tests for tuberculosis; cytomegalovirus (IgM, IgG, and DNA); Human Herpes Virus; Toxopasma (IgM); Rubella (IgM); influenza virus A and B; parainfluenza virus; respiratory syncytial virus; adenovirus; legionella; rickettsia; and Epstein Barr Virus (IgM, IgG and DNA) were all negative. Echocardiography was normal. A diagnosis of acute inflammatory response syndrome and autoimmune vasculitis was entertained on clinical grounds and laboratory findings. Bacterial infection could not be ruled out, then antibiotics (linezolid and cefuroxime) and methylprednisolone was used for 3 days;the high fever subsided and the skin rash disappeared thereafter. 4 days later, high fever recurred and generalized skin eruption was noted. Intravenous immunoglobulin (IVIG) with a dosage of 400 mg/Kg was administered. 8 hours later, his condition deteriorated rapidly to present with a decrease of oxygen saturation, hypotension, tachycardia, tachypnea, irritability and cyanosis. He was transferred to the pediatric intensive care unit (PICU), and administration of fluids, inotropes (dopamine, norepinephrine) and mechanical ventilation was started. Blood routine test showed leukocyte count 10 x 10^9^/L (neutrophil 3 %; eosinophil 40.1 %), erythrocyte count 3.21 x 10^12^/L, hemoglobin 83 g/L, platelet count 46 x 10^9^/L. Total IgE (104.4 KIU/L) was elevated significantly (Table 1). Hypersensitive to IVIG was considered and high-dose intravenous methylprednisolone (30 mg/Kg) was given. During his hospitalization in PICU, the patient experienced several shocks each time after transfusion of blood and blood products, including IVIG, albumin, plasma, washed red blood cells. He developed progressive hepatosplenomegaly, severe pancytopenia (leukocyte count 0.2 x 10^9^/L, erythrocyte count 1.11 x 10^12^/L, hemoglobin 29 g/L, platelet count 9 x 10^9^/L). He presented with neck stiffness, convulsion and light coma, and his eyes couldn't follow light. Serum ferritin (18699 ng/mL), lactate dehydrogenase (1685 IU/L) and triglycerides (4.53 mmol/L) were elevated significantly, but lactic acid was normal and plasma fibrinogen was decreased apparently (0.49 g/L). Both the prothrombin time and the activated partial thromboplastin time were above limit of detection. Lumbar puncture revealed the initial pressure of 125 mmH_2_O and clear cerebrospinal fluid containing white blood cells 0 cells/L, red blood cells 28 cell/L, protein 513 mg/L. The fifth bone marrow aspiration displayed large amount of hemophagocytic histiocytes (Fig. 1). Brain magnetic resonance imaging (MRI) suggested ANEC (Fig. 2). DNA samples were obtained from the peripheral blood of the patient by standard procedures, and the sequences of HLH-associated genes were analyzed. The result verified 1 variation in the *PRF1* gene, 10 variations in the *UNC13D* gene, 8 variations in the *STXBP2* gene, 2 variations in the *XIAP* gene and no variations in *STX11* and *SH2D1A* (Table 2).

**Table 1:** The main laboratory parameters detected in this patient

Laboratory parameters		Reference value	Results
Rheumatoid factor		0–20 IU/ml	<20
Anti-streptolysin “O”		0–200 IU/ml	<25
C-reactive protein		0–10 mg/L	19.5
Anti-cyclic-citrullinated peptide antibody		–	Negative
Autoantibodies	Antinuclear antibody	–	Positive
	Antineutrophil cytoplasmic antiboby	–	Positive
	Anti jo-1 antibody	–	Negative
	Anti-centromere antibody	–	Negative
	Anti-double-stranded dna antibody	–	Negative
	Anti-histone antibody	–	Negative
	Antismith antibody	–	Negative
	Anti-rnp antibody	–	Negative
	Anti-SS A antibody	–	Negative
	Anti-SS B antibody	–	Negative
	Anti-ribosomal antibody	–	Negative
	Anti-Sc1-70 antibody		Negative
	Anti-nucleosome antibody	–	Negative
Lymphocyte subsets (Flow cytometry)	CD3+ T cell	68 % ± 10.7 %	66.1 %
	CD4+ T cell	31.5 % ± 8.8 %	34.2 %
	CD8+ T cell	25.7 % ± 6.5 %	19.66 %
	CD19 B cell	15.6 % ± 5.8 %	19.1 %
	NK cell	5.6 % - 31 %	9 %
	CD4+/CD8+	1.5 ± 0.5	1.74
Ig isotypes	IgG	3.22 – 14.0 g/L	6.39
	IgM	0.57 – 1.41 g/L	0.82
	IgA	0.24 – 1.79 g/L	0.43
	Complement C3	0.8 – 1.6 g/L	0.74
	Complement C4	0.1 – 0.4 g/L	0.18
Total IgE		0 – 20 KIU/L	104.4
Ammonia		9 – 30 umol/L	19
Lactic acid		0.7 – 2.1 mmol/L	1.22
Coagulation test	Activated partial thromboplastin time	26.1 – 40.73 s	29.7^a^, over the upper limit of detection^b^
	Prothrombin time	9.3 –12.9 s	12.8^a^, over the upper limit of detection^b^
	International normalized ratio	0.72 – 1.15	1.09^a^, over the upper limit of detection^b^
	Thrombin time	13.2 – 20.1 s	20^a^, over the upper limit of detection^b^
	Fibrinogen	1.57 – 3.93 g/L	2.43^a^, 0.49^b^
Blood lipid levels	Total cholesterol	3.1 – 5.8 mmol/L	1.78^a^, 3.62^b^
	Triglyceride	0.23 – 1.7 mmol/L	2.54^a^, 4.53^b^
	High density lipoprotein	0.9 – 1.8 g/L	0.47^a^, 1.05^b^
	Low density lipoprotein	2.07 – 4.1 g/L	1.1^a^, 1.8^b^
Serum ferritin		22 – 322 ng/ml	15.4^a^, 18699^b^
Values of eosinophils		0.02 – 0.5 × 10^9^/L	0.26^a^, 4.01^c^, 0.01^d^

Note: ^a^ - on admission, ^b^ - peak period of disease, ^c^ - the 2nd day after first IVIG, ^d^ - when severe pancytopenia

**Table 2:** Variations of the HLH related genes and the references [34]

Gene	Exons	Function	Variation	rs number	MAF	Clinical significance
PRF1	EXON3	Synonymous	c.900C > T p.His300His	rs885822	0.3041	
UNC13D	EEXON1	Intron	c.117 + 59C > T	rs3744010	0.2847	
	EXON4	Synonymous	c.279C > T p.Pro93Pro	rs3744007	0.1000	
	EXON5	Intron	c.388 + 122C > T	rs3744006	0.4930	
	EXON11	Synonymous	c.888G > C p.Pro296Pro	rs7223416	0.4902	
	EXON18	Intron	c.1596 + 36A > G	rs3744026	0.3083	
	EEXON19	Intron	c.1728-48 T > C	rs3744024	0.3025	
	EXON21	Synonymous	c.1977C > T p.Thr659Thr	rs2290770	0.0799	
	EXON24	Intron	c.2299-46C > T	rs7212635	0.2542	
	EXON28	Intron	c.2709 + 48C > T	rs2290768	0.2530	
	EEXON32	Synonymous	c.3198A > G.Glu1066Glu	rs7210574	0.4738	
STX11	No mutation					
STXBP2	Exon2	Intron	c.38-7C > T	rs113939878	NA	acceptor
	Exon10	Synonymous	c.816 C > T p.Ser272Ser	rs78010345	0.0034	
	EXON15	Intron	c.1247-43 T > C	rs929807	0.4730	
	EXON15	Intron	c.1356 + 18A > G	rs889187	0.4836	
	Exon16	Synonymous	c.1443 T > C .Asp481Asp	rs10001	0.4958	
	Exon18	Intron	c.1696 + 28G > C	rs34976997	0.3157	
	Exon18	Intron	c.1696 + 77G > A	rs794074	0.4615	
	Exon19	Intron	c.1697-26 T > G	rs794073	0.465	
XIAP	Exon5	Intron	c.1099 + 264G > C	rs28382732	0.2638	
	Exon7a	3' UTR	c.*12A > G	rs28382740	0.2673	
SH2D1A	No mutation					

Note: *MAF* - minor allele frequency; *UTR*, untranslated region

The diagnosis of hemophagocytic lymphohistiocytosis and acute necrotizing encephalopathy was made based on the above findings. The HLH-2004 treatment protocol was used once diagnosis made. The clinical symptoms were rapidly improved. Followed up to 3½ years old, he was well. The neurological image (MRI) showed slightly abnormal shape of bi-lateral ventricular, not any other obvious abnormal noticed.

## Discussion

HLH may occur either as a primary condition with a genetic mutation or as a secondary condition associated with an infection, immunologic disorder, malignancy, or metabolic disease [6–8]. This patient presented with persistent fever, skin rash, ensuing hypersensitive to all blood products, hepatosplenomegaly, pancytopenia, and central nervous symptoms. He fulfilled 5 of the 8 criteria for acquired HLH [9]. Hemophagocytosis is one of the criteria and reported to be negative at diagnosis in about 20-25 % of cases as reported before and more recently in a case series [10, 11]. As some researcher reported that the repetitive bone marrow aspirations may reveal hemophagocytosis in the further course [12]. No hemophagocytosis was detected in the first four bone marrows in this case, but the fifth bone marrow biopsy displayed large amount of hemophagocytic histiocytes. It is important to note that the diagnosis of HLH does not depend on this morphological finding. The genes relevant to familial HLH (FHLH) including *PRF1*, *UNC13D*, *STX11*, *STXBP2* [3], were analyzed to confirm the mutations, as well the genes related to immune deficiency syndromes associated with HLH: *XIAP* and *SH2D1A* [3, 13]. Sequencing results showed that all these variations were synonymous or in intron region and untranslated region. Analysis of the genetic data revealed that no variation was likely to produce modified amino acid sequence, thus to cause a severe impairment of protein function in this patient, with addition of the fact that there was no HLH patient or consanguinity in his family. This information is therefore relevant in that it is not likely to be genetic HLH.

This patient presented with neck stiffness, convulsion and light coma; and the severe lesions of brain MRI showed changes mainly in thalamus, basal ganglia, brainstem and white matter as described in patients with ANEC [14, 15], which is characterized by multiple and symmetrical lesions with edema and necrosis in the thalamus, the cerebral and cerebellar medulla, and the brainstem tegmentum [4, 16], which evoked the diagnosis of acute necrotizing encephalopathy of childhood. The clinical feature of typical ANEC is fulminant, with severe neurologic sequelae and profoundly high mortality [17]. However, the discrepancy between the severe MRI findings, the relatively mild neurological manifestations, and the unexpected clinical outcome was not consistent with ANEC. We speculated that the brain damage was not true ANEC, but it was caused by transient ischemia induced by allergic vasculitis due to hypersensitivity. The extensive allergic vasculitis involves central nerve system, and extravasation of plasma takes place secondary to local vascular lesions. This is followed by destruction and necrosis of glial cells and neurons [18]. The neurological signs were improved promptly with the subsiding of hypersensitivity, which gave a good interpretation to our speculation.

The central nervous system (CNS) involvement was reported in about a third of the children with HLH, which could easily be misdiagnosed in many children because its radiologic manifestations are nonspecific and overlap with various infectious and inflammatory disorders. Therefore, differential diagnosis of resembling diseases is very important for this patient. A wide range of disorders should be considered in the differential diagnosis, including Leigh disease, hypoxic-ischemic encephalopathy, viral encephalitis, hemorrhagic encephalitis, acute disseminated encephalomyelitis, and Reye syndrome. First of all, the brain damages focused on thalami and brainstem was not consistent with CNS involvement in HLH [18]. Absence of inflammatory cells in cerebrospinal fluid and the symmetric brain lesions in this case differentiates from the entities of acute disseminated encephalomyelitis and acute hemorrhagic encephalitis. Reye syndrome can be excluded by hyperammonemia, hypoglycemia, and lactic acidosis absent in this case [19], as well as Leigh disease. The differentiation from hypoxic-ischemic encephalopathy presented much more difficulty in this patient. In spite of shocks, the oxygen saturation, heart rate and blood pressure were corrected quickly, which did not influence cerebral perfusion severely. Moreover, the imaging findings could not explain hypoxic-ischemic brain damage which focuses on obscuration of grey-white matter junctions and widespread laminar necrosis of the cortex [20]. Furthermore, certain viral encephalitis may be difficult to exclude clinically e.g. Japanese encephalitis may also involve the deep gray matter symmetrically. However, the involvement of the brain stem is uncommon in Japanese encephalitis, and thalamic involvement is not necessarily symmetrical [21].

The etiologies of acquired HLH and ANEC were suggested to be associated with infectious, metabolic, immune related causes [6, 7, 15], and hypersensitivity was more recently reported to be complicated by hemophagocytic lymphohistiocytosis in a case series [22–24]. This boy did not meet the diagnostic criteria of systemic lupus erythematosus [25], and there was no arthritis during the disease course so he was not consistent with the diagnosis of juvenile idiopathic arthritis [26]; moreover, there was not any evidence of scleroderma and mixed connective tissue disease, though he was detected anti-nuclear and anti-neutrophil auto-antibodies and severe pancytopenia. He suffered allergy in his past and had positive allergy family history; eosinophils and IgE significantly elevated after IVIG and blood transfusion then gradually declined followed steroids; furthermore, he was hypersensitive to all kinds of blood and blood products, and subsequently, developed cytopenias. All above, it led us to speculate that allergic reaction was involved in the pathogenesis of HLH and brain lesions in this patient. This was never reported previously.

Increase of eosinophils and reaction to blood products suggests type I, II and III hypersensitivity was involved in the pathogenesis in this case. Hypersensitivity and HLH appear somewhat similar, being characterized by activated lymphocytes and hypercytokinemia [27]. Hypersensitivity results in hypercytokinemia, leading to uncontrolled activation of benign scavenger macrophages and development of hemophagocytosis [22]. The pathogenesis of HLH involves the dysfunction of natural killer (NK) cells and cytotoxic T cells leading to prolonged and intense activation of antigen-processing cells (macrophages and histiocytes) [28] and CD8+ T cells, and excessive proliferation and ectopic migration of T cells, which triggers overproduction of proinflammatory cytokines, and unrestrained hemophagocytosis [29]. It is suggested that cell destruction by cytotoxic antibodies and a reversible depression of stem cell activity with myeloid maturation blockade contribute to the pathophysiology of the cytopenias. The deposited immune complexes can trigger neutrophils to release free radicals and enzymes causing tissue destruction, which can lead to endothelial cell necrosis [30]. Hypercytokinemia and the hyperpermeability of both the blood-brain barrier and the capillary walls of the central nervous system might be essential in the pathogenesis of acute necrotizing encephalopathy [31].

Hemophagocytic lymphohistiocytosis has a poor prognosis [32]. Patients with secondary HLH have had only a 55 % survival at 3 years [33]. This boy was followed up regularly after continuation therapy. Followed up to 3½ years old, he is doing well; there is not any symptom that could be due to central nervous system dysfunction; the routine blood count, blood lipid level, liver function and ferritin were normal, and the bone marrow cytology demonstrated sustained remissions. The neurological image (MRI) showed no obvious abnormal.

## Conclusions

This is a rare case of hemophagocytic lymphohistiocytosis, with specific presentation resembling ANEC. Hypersensitivity may involve in the etiologies of HLH and brain damage, which have good clinical and neurologic outcome if treated early. From this study, we should have the awareness of such a rare disease as delay of diagnosis and treatment is common with subsequent poor outcome. Early diagnosis is crucial to reduce mortality rates. It is of importance to know that hypersensitivity may result in HLH and brain damage resembling ANEC from the findings in this study.

## Abbreviations

ANEC, acute necrotizing encephalopathy of childhood; CNS, central nervous system; DNA, deoxyribose nucleic acid; HLH, hemophagocytic lymphohistiocytosis; Ig, immunoglobulin; IVIG, intravenous immunoglobulin; MRI, magnetic resonance imaging; NK, natural killer; PICU, pediatric intensive care unit
